# Recurrent spontaneous coronary artery dissection, diagnostic issues, and management: a case report

**DOI:** 10.1093/ehjcr/ytag529

**Published:** 2026-07-18

**Authors:** Nicolas Combaret, Clément Quinonero, Pascal Motreff, Géraud Souteyrand

**Affiliations:** Department of Cardiology, University Hospital of Clermont-Ferrand, 58 rue Montalembert, Clermont-Ferrand 63000, France; Department of Cardiology, University Hospital of Clermont-Ferrand, 58 rue Montalembert, Clermont-Ferrand 63000, France; Department of Cardiology, University Hospital of Clermont-Ferrand, 58 rue Montalembert, Clermont-Ferrand 63000, France; Department of Cardiology, University Hospital of Clermont-Ferrand, 58 rue Montalembert, Clermont-Ferrand 63000, France

**Keywords:** Spontaneous coronary artery dissection, Acute coronary syndrome, Acute myocardial infarction, Case report

## Abstract

**Background:**

Spontaneous coronary artery dissection (SCAD) is a severe and under-diagnosed pathology that generates diagnostic and therapeutic difficulties.

**Case summary:**

This case is about a 45-year-old female with a history of coronary spasm in 2012 without ergonovine provocation confirmation. She was admitted in 2014 for an acute coronary syndrome (ACS) revealing an aspect of thrombus of the left anterior descending (LAD) coronary artery. GPIIb/IIIa antagonists, unfractionated heparin, and antiplatelet therapy were introduced. Angiographic control 10 days later showed that the lesion was not thrombotic but a SCAD which spread on the left main coronary artery and LAD proximal part. Angiographic control at 1 month showed a partial healing but persistent focal dissection of the mid-LAD treated by two bioresorbable vascular scaffolds. In 2018, she presented with a new case of SCAD treated at another hospital, where she had been misdiagnosed with myocarditis. A review of the angiograms revealed findings typical of distal SCAD of the circumflex artery. In June 2022, a new ACS was related to a right coronary artery SCAD. The distal part was occluded by an extensive haematoma. This was turned into a dissection by using a cutting balloon. No stent was implanted.

**Discussion:**

SCAD is a complex condition that presents diagnostic challenges, as this case illustrates. Such diagnostic errors can lead to the prescription of inappropriate medication, such as anticoagulant therapy, which may exacerbate the condition by increasing the size of the haematoma. It is also a condition that can recur, and clinicians must bear this in mind.

Learning points

- Spontaneous coronary artery dissection (SCAD) should be suspected in case of acute myocardial infarction in young women with no or few cardiovascular risk factors, especially in the case of a previous episode of SCAD.
- Anticoagulants should be avoided in SCAD management because it can worsen lesions, especially in the case of wall haematomas.

## Introduction

Spontaneous coronary artery dissection (SCAD) is a severe and under-diagnosed disease.^1^ It causes acute coronary syndrome (ACS) that differs from atherosclerotic aetiology by their physiopathology, presentation, and treatment. Although this pathology was thought to be rare, it is becoming more and more common with the increased use of early angiography in management of acute chest pain.^2^ This diagnosis is challenging and needs expert knowledge and experience. The aim of this case report is to illustrate the diagnostic and therapeutic difficulties that physicians may encounter in dealing with this pathology.

## Summary figure

**Figure:**
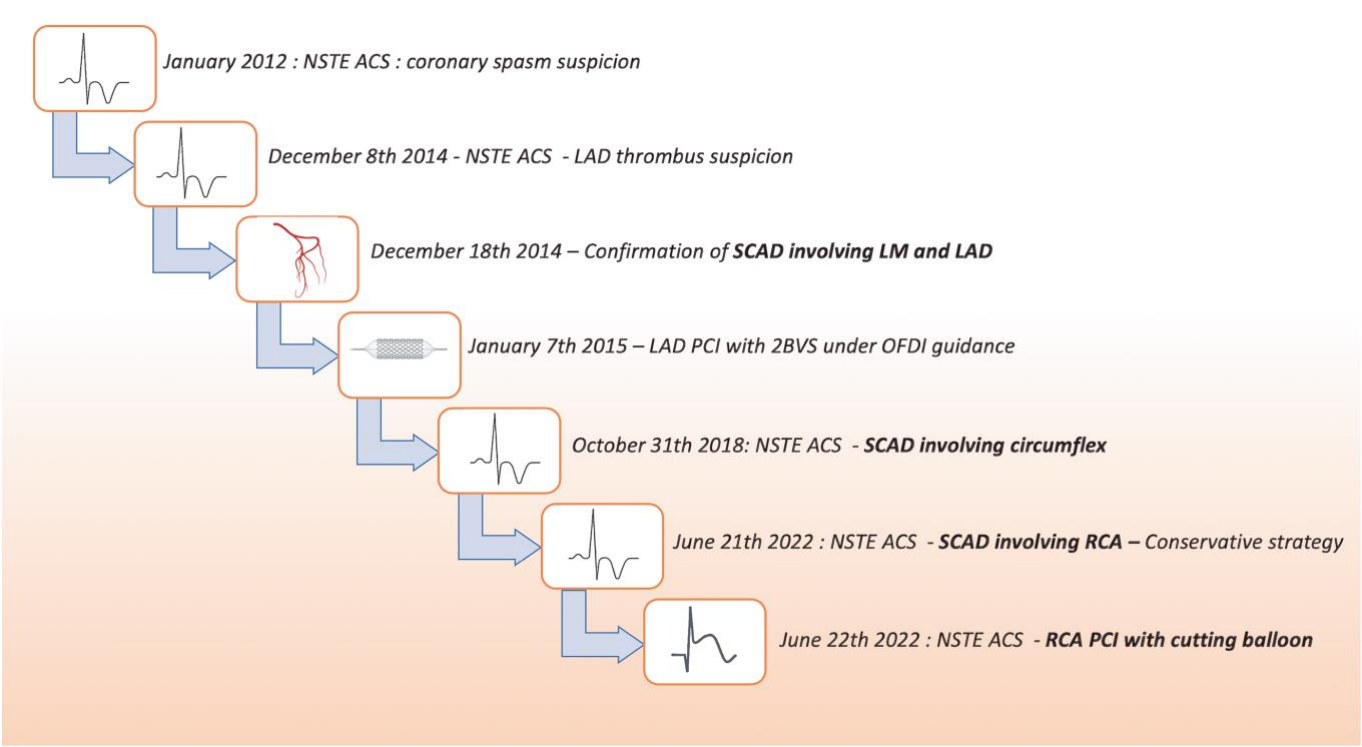


## Case presentation

This case is about a 45-year-old Caucasian woman with an unremarkable medical history. She had no cardiovascular risk factors except for a past cigarette smoking intoxication.

In 2012, she suffered an ST elevation myocardial infarction (STEMI) with angiographically normal coronary arteries. Diagnosis of coronary spasm was reached without intravenous ergonovine provocation. Her long-term medication consisted of amlodipine, low dose aspirin, and statins. Her contraception was an intrauterine contraceptive device liberating progesterone.

**Figure 1:**
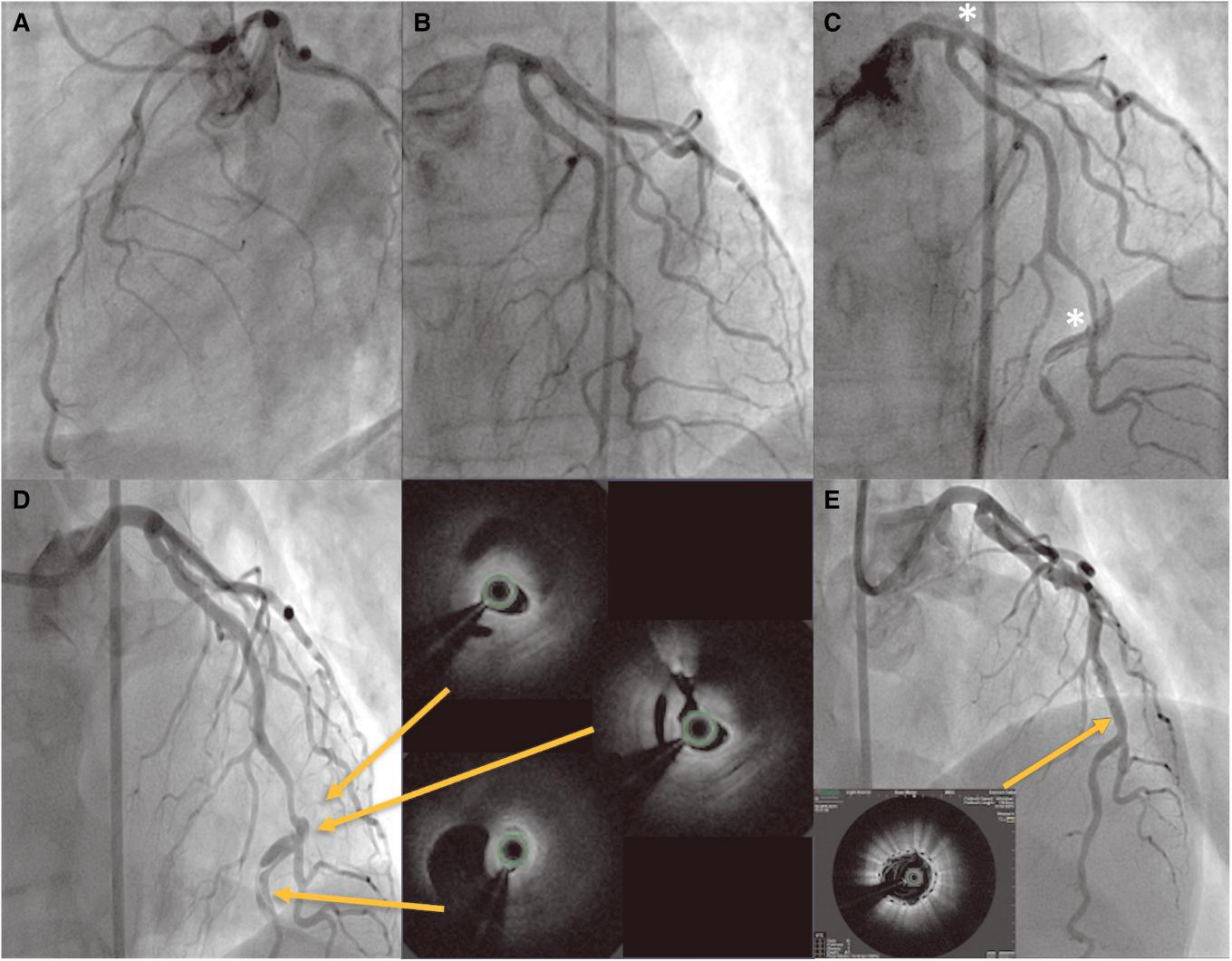
2014 angiograms showing the mid-part of left anterior descending stenosis suggesting a coronary thrombus (*A* and *B*). Systematic control at day 10 with an extensive dissection involving the left anterior descending and the left main (*) (*C*). Systematic control at 3 weeks after anticoagulation stop with healing of the left main coronary artery but persistent left anterior descending dissection analysed by OFDI (*D*). Angiographic and OFDI result after stenting with 2 BVS (*E*).

In December 2014, she was admitted to another hospital for chest pain with troponin elevation and without electrocardiographic modification. Early coronary angiography revealed a focal stenosis without occlusion (TIMI 3 flow) in the median part of the left anterior descending (LAD) artery due to a focal dissection (*Figure 1 A-B*). This dissection was misdiagnosed and the diagnosis of coronary thrombus was retained. It was decided not to perform percutaneous coronary intervention (PCI) but to promote medical treatment with aspirin, clopidogrel, and GPIIb/IIIa antagonists during 24 h relayed by unfractionated heparin with the wrong aim to quickly melt the clot. Systematic angiographic control 10 days later corrected the diagnosis and showed an extensive haematoma of the left main coronary artery and a worsening of the LAD dissection (*Figure 1 C*). Due to the lack of symptoms or occlusive lesion, no stent was implanted. It was decided to stop heparin. Statins were empirically prescribed despite the absence of the atheromatous lesion at coronary angiography. β-Blockers have been introduced with the aim of preventing recurrence. The patient was referred to our institution for subsequent care. After 21 days of in-hospital monitoring, the angiographic control revealed complete healing of the left main coronary artery, but persistent mid-LAD dissection. Optical frequency domain imaging (OFDI) confirmed that the wire was in the true lumen (*Figure 1D*). It was decided to treat this persistent dissection by the implantation of two bioresorbable stents. The angiographic and OFDI results were satisfactory (*Figure 1E*). Her medical treatment at discharge consisted of bisoprolol, atorvastatin, and aspirin combined with clopidogrel for 1 year.

**Figure 2:**
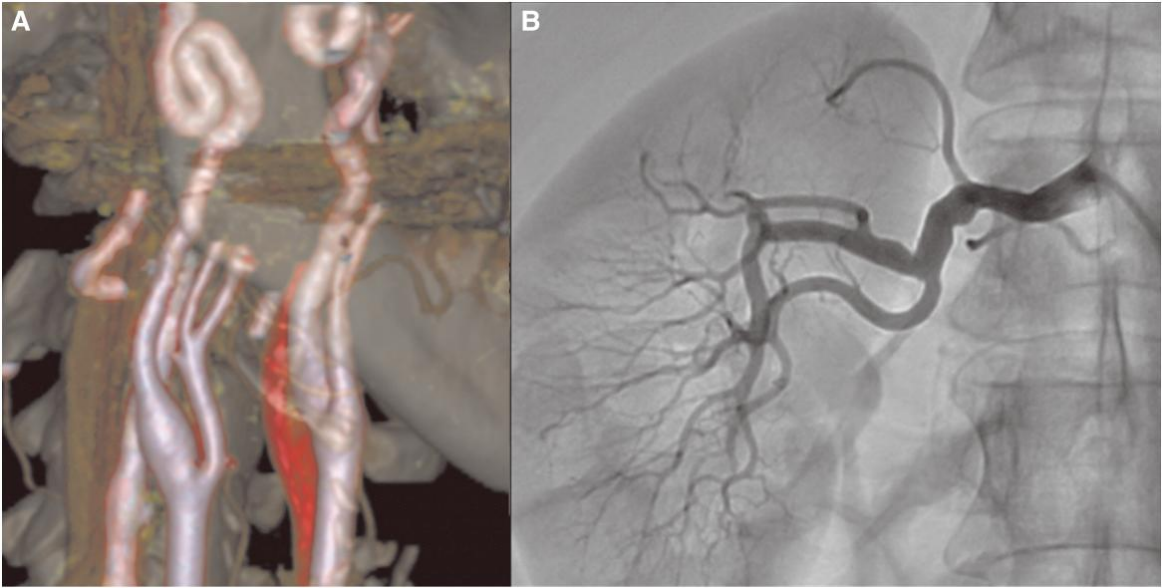
Computed tomography reconstruction of supra-aortic trunks with fibro-muscular dysplasia of internal carotids (*A*); right renal artery angiography with signs of fibro-muscular dysplasia (*B*).

An aetiologic assessment was performed and found no argument for a connective tissue disease such as Marfan or Ehlers– Danlos syndromes. The link between SCAD and these conditions has long been recognized, but it is in fact a relatively rare association (<10%).^3^ Magnetic resonance imaging (MRI) and computed tomography (CT) showed evidence of fibro-muscular dysplasia of the internal carotid arteries and the right vertebral artery. An angiography showed incipient fibro-muscular dysplasia of the right renal artery. The left renal artery and iliac arteries were not damaged (*Figure 2*).

**Figure 3:**
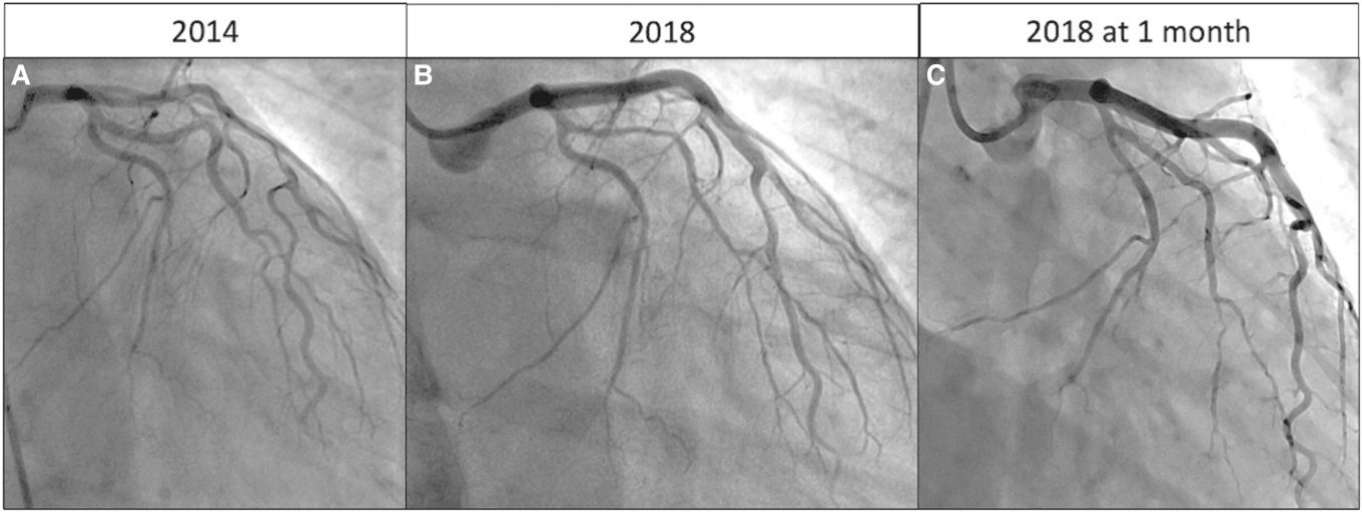
Angiographic evolution of the marginal branch before and during the 2018 episode. Normal in 2014 (*A*); compressive dissection of the marginal branch during acute coronary syndrome (*B*); healing at 1 month with persistence of distal haematoma (*C*).

In October 2018, the patient suffered from another ACS with negative T waves on DI/aVL. Troponin elevation confirmed the recurrence of non-ST elevation myocardial infarction. A coronary angiography found no abnormalities on the LAD artery scaffolds. There was an elongated but insignificant stenosis of the first marginal branch. Cardiac MRI showed perfusion disturbances and late enhancement on laterobasal wall consistent with the diagnosis of myocarditis. Diagnosis of first marginal branch dissection was made after angiography proofreading in a reference centre. A 1-month angiographic control revealed healing of the proximal part of this marginal branch with probable residual haematoma in the distal part (*Figure 3*).

Despite a medical treatment with beta-blockers and aspirin, she suffered another ACS in June 2022 with syncope but no EKG modification. A coronarography revealed a wall haematoma of the right coronary artery (RCA) terminal part and the posterior descending artery (PDA) without slow flow (TIMI 3). Medical management was preferred to angioplasty.

**Figure 4:**
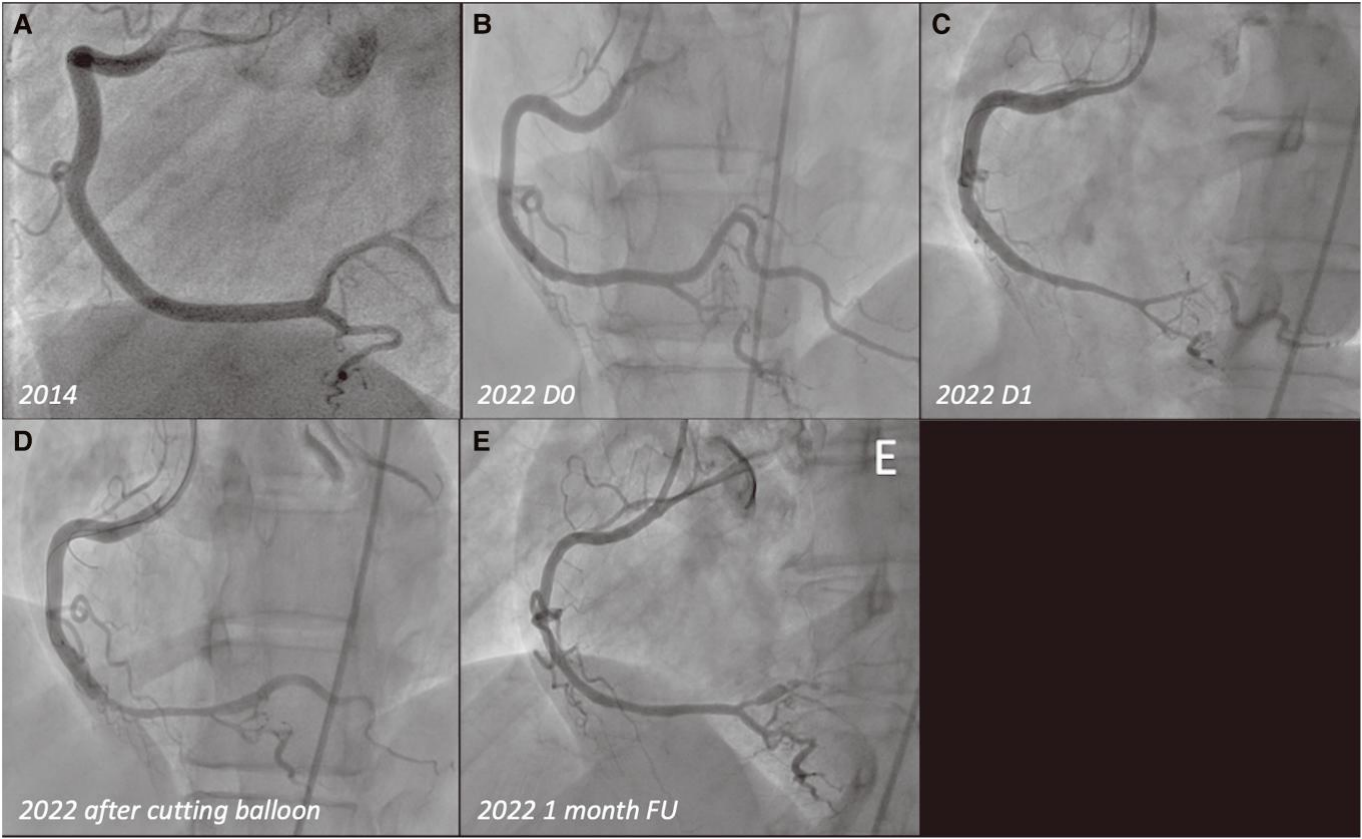
Angiographic evolution of the right coronary artery. Normal in 2014 (*A*); distal right coronary artery dissection with extension to the posterior descending artery at day 0 of acute coronary syndrome in 2022 (*B*); worsening with ST elevation and posterior left ventricular branch/posterior descending artery occlusive dissection at day 1 in 2022 (*C*); improved circulation in the posterior left ventricular branch after cutting balloon (*D*); healing of the distal right coronary artery and posterior descending artery at 1 month control but persistent posterior left ventricular branch occlusion (*E*).

The next day, an ST elevation appeared in inferior territory with recurrent pain. The haematoma was growing with sub-occlusion of the RCA terminal segment. A PCI with cutting balloon was performed to transform this haematoma into a dissection, allowing a recovery of the flow and a complete regression of the pain. No stent was implanted. A planned control at 1 month showed a healing of the distal part of RCA dissection with persistent posterior left ventricular branch (PLV) occlusion (*Figure 4*).

## Discussion

In the present article, we present a case of recurrent spontaneous coronary artery dissection that highlights diagnostic and therapeutic issues regarding this pathology.

Firstly, this case highlights the angiographic challenges inherent in the diagnosis of SCAD. Misdiagnosis is common and confusion with beginning atherosclerosis or coronary spasm is common.^1^ Angiographic signs like radiolucent flap, contrast dye staining of the arterial wall, or long narrowing of lumen diameter (‘stick insect’ or ‘radish’ aspect) help practitioners with SCAD detection.^4^ Intravascular imaging techniques can also be useful.

It is important to consider clinical characteristics of patients admitted for acute myocardial infarction. According to the literature, the mean age of patients with SCAD is 51.5 years, mostly women (90.6%), and 54.7% of cases had less than two cardiovascular risk factors.^2^ A French series reported SCAD in 36% of women under 60 years with ACS and one or fewer cardiovascular risk factors.^4^ This is why, as in this case, one must bear in mind the possibility of SCAD in young women with no or few cardiovascular risk factors who suffer from ACS.

Furthermore, this patient has presented four coronary events on three different arteries in 10 years. The recurrence rate of SCAD is approximately 3.3% at 1 year in a French cohort^2^ and 10.4% at 3.1 years^5^ in the Canadian registry. In case of new acute myocardial infarction, a history of SCAD should primarily suggest a recurrence. Prevention of recurrence includes control of hypertension, antiplatelet therapy, and beta-blockers.^5^ Recent data from the Australian and New Zealand registry suggest that recurrences are more common in case of dual antiplatelet therapy with ticagrelor, previous stroke, and presence of FMD.^6^

Secondly, this case report focuses on the harmful effects of anticoagulants on spontaneous coronary haematomas evolution. Current data plead for anticoagulants and GPIIb/IIIa antagonists eviction for the management of SCAD.^7^ Indeed, taking into account the pathophysiology of SCAD (more specifically the ‘outside-in’ theory, it is thought that a spontaneous haematoma initially develops, causing compression of the arterial lumen, which subsequently progresses to a true dissection due to the occurrence of an intimal rupture), it seems logical to limit the use of any treatment that promotes the progression of the haematoma, such as anticoagulant therapy or GPIIb/IIIa antagonists. In the case of PCI with stent implantation, dual antiplatelet therapy remains mandatory (with clopidogrel) for 12 months as with every ACS; but in the case of conservative therapy, it could be preferable to maintain only simple antiplatelet therapy by aspirin. The last ESC report recommends double antiplatelet treatment after ACS for 1 year, but some papers seem to favour single antiplatelet treatment in first intention in the context of SCAD conservatively treated.^8^

Finally, this case report highlights the known association between SCAD and fibro-muscular dysplasia which has been described since 2010 but has shown a highly variable association rate in the literature due to the lack of systematic screening in studies.^7, 9^ The link between these two conditions has not been clearly established, even though there appear to be identical genetic factors involved.^10^

## Data Availability

Additional anonymized data are available from the corresponding author upon reasonable request.

## Lead author biography

Dr N. Combaret is a French interventional cardiologist. His areas of research include acute coronary syndrome, in particular spontaneous coronary artery dissection, and intra-coronary imaging.
